# *Hizikia fusiformis*: Pharmacological and Nutritional Properties

**DOI:** 10.3390/foods10071660

**Published:** 2021-07-19

**Authors:** Maria Dyah Nur Meinita, Dicky Harwanto, Jae-Hak Sohn, Jin-Soo Kim, Jae-Suk Choi

**Affiliations:** 1 Seafood Research Center, Industry Academy Cooperation Foundation (IACF), Silla University, 606, Advanced Seafood Processing Complex, Wonyang-ro, Amnam-dong, Seo-gu, Busan 49277, Korea; dickyharwanto@lecturer.undip.ac.id (D.H.); jhsohn@silla.ac.kr (J.-H.S.); 2 Faculty of Fisheries and Marine Science, Jenderal Soedirman University, Purwokerto 53123, Indonesia; 3 Center for Maritime Bioscience Studies, Jenderal Soedirman University, Purwokerto 53123, Indonesia; 4 Faculty of Fisheries and Marine Science, Diponegoro University, Semarang 50275, Indonesia; 5 Department of Food Biotechnology, College of Medical and Life Sciences, Silla University, 140, Baegyang-daero 700 beon-gil, Sasang-gu, Busan 46958, Korea; 6 Department of Seafood and Aquaculture Science, Gyeongsang National University, 38 Cheongdaegukchi-gil, Tongyeong-si 3064, Korea

**Keywords:** *Hizikia fusiformis*, *Sargassum fusiforme*, hijiki, nutritional properties, pharmacological properties

## Abstract

The brown seaweed *Hizikia fusiformis* (syn. *Sargassum fusiforme*), commonly known as “Hijiki”, has been utilized in traditional cuisine and medicine in East Asian countries for several centuries. *H. fusiformis* has attracted much attention owing to its rich nutritional and pharmacological properties. However, there has been no comprehensive review of the nutritional and pharmacological properties of *H. fusiformis*. The aim of this systematic review was to provide detailed information from the published literature on the nutritional and pharmacological properties of *H. fusiformis*. A comprehensive online search of the literature was conducted by accessing databases, such as PubMed, SpringerLink, ScienceDirect, and Google Scholar, for published studies on the nutritional and pharmacological properties of *H. fusiformis* between 2010 and 2021. A total of 916 articles were screened from all the databases using the *preferred reporting items for systematic reviews and meta-analyses* method. Screening based on the setdown criteria resulted in 59 articles, which were used for this review. In this review, we found that there has been an increase in the number of publications on the pharmacological and nutritional properties of *H. fusiformis* over the last 10 years. In the last 10 years, studies have focused on the proximate, mineral, polysaccharide, and bioactive compound composition, and pharmacological properties, such as antioxidant, anticancer, antitumor, anti-inflammatory, photoprotective, neuroprotective, antidiabetic, immunomodulatory, osteoprotective, and gastroprotective properties of *H. fusiformis* extracts. Overall, further studies and strategies are required to develop *H. fusiformis* as a promising resource for the nutrition and pharmacological industries.

## 1. Introduction

*Hizikia fusiformis* (Harvey) Okamura, commonly known as “hijiki seaweed” (syn. *Sargassum fusiforme* (Harvey) Setchell), is an edible brown alga belonging to the class Phaeophyceae, order Fucales, and family Sargassaceae. *H. fusiformis* grows naturally on lower intertidal rocks around the coastline of the northwest Pacific Ocean [1], and is widely used as a food delicacy, marine vegetable, and medicinal herb in China, Korea, Japan, and Southeast Asia [2]. Currently, *H. fusiformis* has been effectively cultivated in southern China and Korea. In 2015, South Korea produced 28,157 tons of *H. fusiformis* dry weight (DW) ha^−1^ year^−1^ with a value of USD 15,227,000, making South Korea one of the largest producers of Hizikia worldwide [3].

The demand for *H. fusiformis* has increased owing to its nutritional potential as well as to its economic value in the pharmaceutical and manufacturing industries [4]. Most of the existing studies on *H. fusiformis* have mainly focused on the industrial use of its hydrocolloid; however, recent studies have examined the medical efficacy of *H. fusiformis*. Several compounds such as polysaccharides, fucoidan, fucosterol, and phenols with various pharmacological properties have been isolated and identified from *H. fusiformis* [4,5,6,7]. These bioactive compounds show anti-inflammatory [8], antioxidant [9], antitumor [10], immunomodulatory [11], and protective effects against osteo-disease [12,13]. Among the various bioactive compounds found in seaweed, phenolic compounds have attracted the most interest. Some comprehensive studies have investigated phenolic compounds of terrestrial plants, including their antioxidant properties [14,15]. Humans need antioxidants to prevent the formation of free radicals and reactive oxygen species (ROS), which can cause lipid peroxidation and cell damage. Seaweed, as a benthic marine organism that can survive in harsh and exposed environments, produces high concentrations of phenolic compounds in the form of phlorotannin as an antioxidant source. The phlorotannin and phenolic compounds in seaweed can potentially be used in reducing oxidative reactions in biological systems [16,17,18]. For instance, the hydrophilic phlorotannins extracted from *H. fusiformis* showed significantly higher radical scavenging activities than did those from original extracts and can be developed as a great source of natural antioxidative nutraceuticals [19]. However, compared with those from terrestrial plants, phenolic compounds from seaweed have not been studied extensively, and no comprehensive review papers regarding the pharmacology and nutritional properties of *H. fusiformis* have been published. Therefore, the aim of this systematic review was to provide detailed information from the published literature on the nutritional and pharmacological properties of *H. fusiformis*.

## 2. Materials and Methods

**Figure 1:**
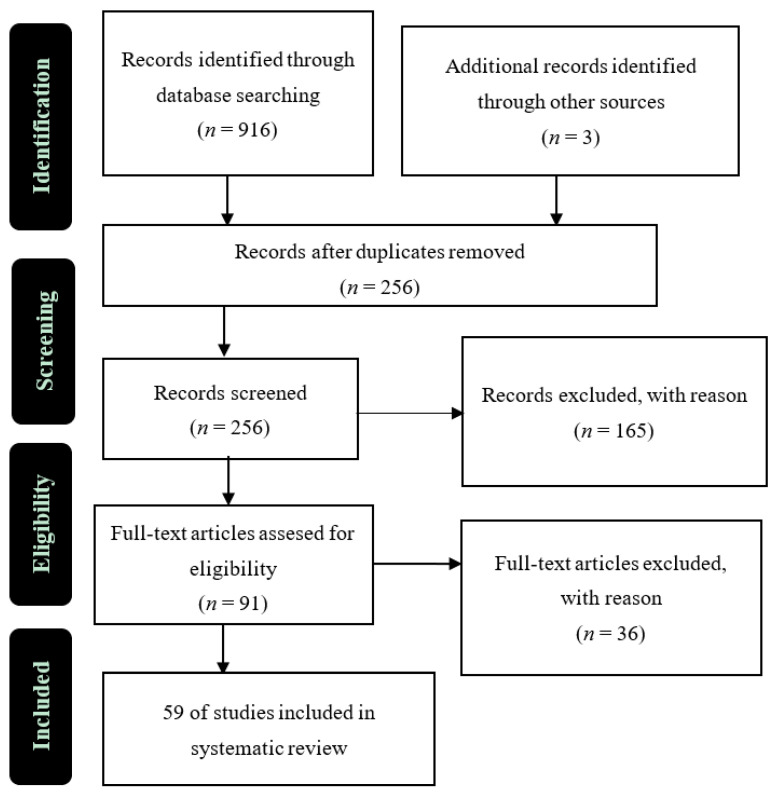
Summarized search method based on the PRISMA method.

We conducted a systematic literature review of the published studies on the pharmacological properties of *H. fusiformis*. The literature was analyzed using the Preferred Reporting Items for Systematic reviews and Meta-Analyses (PRISMA) method [20] (Figure 1).

A comprehensive online search of the literature was conducted by accessing the following databases: PubMed, SpringerLink, ScienceDirect, and Google Scholar, for studies published between 2010 and 2021. We used the keyword “*Hizikia fusiformis*”, “*Sargassum fussiforme*”, and “hijiki” combinations in the title, abstract, or text content. The complete list of identified articles was maintained using Mendeley’s open-source reference management (https://www.mendeley.com/ (accessed on 1 March to 18 July 2021)).

Articles obtained from the database search using the search criteria above were aggregated in the second stage, and duplicate articles were removed. Furthermore, article screening was conducted by reading the ‘title’ and then the ‘abstract’. Studies that did not meet the inclusion criteria were excluded. In the final stage, the remaining articles were screened by reading the full text, and articles that did not meet the inclusion criteria were excluded. The remaining articles were analyzed and extracted, and data were presented in tables and graphs.

## 3. Results

**Figure 2:**
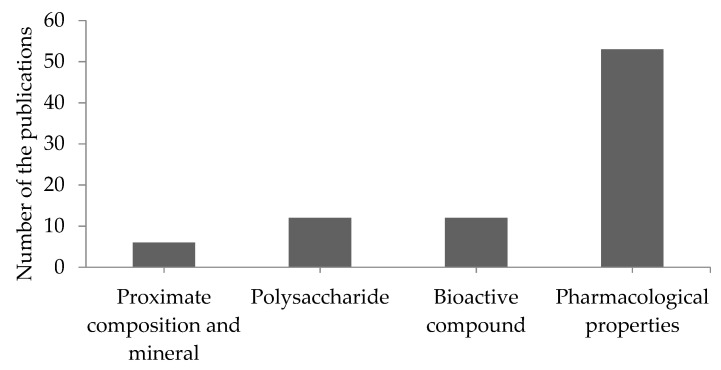
The number of publications related to the nutritional and pharmacological properties of *H. fusiformis*.

A total of 916 articles were screened from all the databases using the PRISMA method. Three more publications were discovered by manually scanning the reference lists. After the selection process, a total of 59 articles were analyzed and are summarized in Figure 2. Among the 59 articles included in the study, seven examined the nutritional properties (proximate composition, major mineral, trace element, and polysaccharide) of *H. fusiformis*, 29 examined its pharmacological properties, and 24 examined both topics. Studies on the pharmacological and nutritional properties of *H. fusiformis* between 2010 and 2021 are summarized in Figure 2.

**Figure 3:**
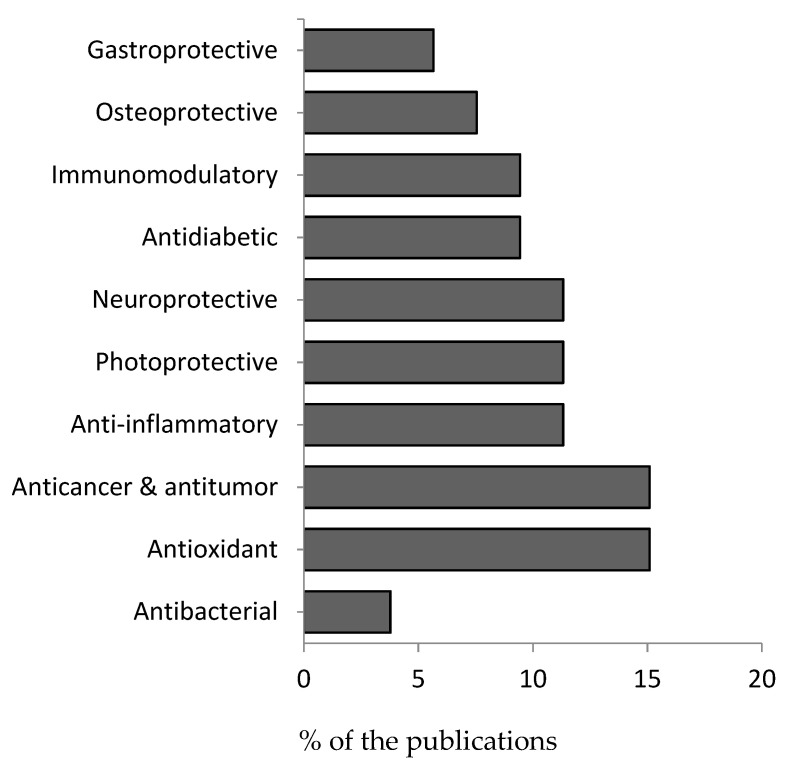
Current research trends of the pharmacological properties of *H. fusiformis*.

Between 2010 and 2021, published articles on the pharmacological properties of *H. fusiformis* focused on the antibacterial (3.77%), antioxidant (15.09%), anticancer and antitumor (15.09%), anti-inflammatory (11.32%), photoprotective (11.32%), neuroprotective (11.32%), antidiabetic (9.43%), immunomodulatory (9.43%), osteoprotective (7.55%), and gastroprotective (5.66%) properties of *H. fusiformis* (Figure 3).

### 3.1. Nutritional Properties

Seaweed is a leading material in the production of new medicines, food formulations, and cosmetics because it contains several important macronutrients such as proteins, carbohydrates, and minerals. Additionally, seaweed is a rich source of health-promoting secondary metabolites, such as phenols, flavonoids, alkaloids, and tannins, with a wide range of applications in the treatment of several disorders and diseases.

#### 3.1.1. Proximate Composition

The proximate composition of *H. fusiformis*, including total carbohydrates, crude protein, crude lipid, crude fiber, ash content, and moisture content, is presented in Table 1. 

The primary metabolites of *H. fusiformis* are essential for its survival, growth, and proliferation. In terms of nutrition, *H. fusiformis* has been used as a food due to its high nutrient value and low calorific content. The carbohydrate content is high (average of 50% of its dry weight); however, a large percentage is in the form of dietary fibers, which are undigestible by humans. Moreover, dietary fibers are beneficial to human health because of their positive effects on the intestinal environment [27,28]. The total carbohydrate content of *H. fusiformis* ranged from 40.73–61.85% DW [21,23,24], which is similar to the carbohydrate content of brown seaweeds (12.2–56.4% DW) reported in previous studies [27,29].

The protein content of *H. fusiformis* ranged from 9.9–18.41% DW. Brown seaweeds, especially the genus Sargassum, have low protein contents (9–20% DW) compared to other seaweed groups [28] and terrestrial vegetable protein sources, such as soybean (40% DW) [30]. Despite their low protein content, seaweeds may be considered potential sources of protein for human and animal nutrition because protein is the building block of living tissues and organs, making it essential in human and animal diets [29].

The lipid content of *H. fusiformis* ranged from 1.2–1.76% DW. Seaweeds are generally low in lipids (0.1–4.5% DW) [31,32]. Although the lipid content of seaweed is low, it is important to differentiate them based on their chemical characteristics [29]. Polyunsaturated fatty acids (PUFAs), derived from algae, have been shown to play important roles as an energy source and in cell-membrane components [31].

The fiber content of *H. fusiformis* reported in this review (11.3% DW) was higher than that of *Laminaria japonica*, *Porphyra tenera*, *Undaria pinnatifida*, and *Palmaria palmata* (6.5–8.0% DW). Thus, confirming the results of previous studies that reported higher fiber content for brown seaweed compared with that of red and green seaweed [29]. Generally, the daily dietary fiber level of human diet is low [33], making *H. fusiformis* an alternative and sustainable dietary fiber source [34,35]. Although some seaweed-derived fibers (alginate, carrageenan, and agar) have been used for years to enhance the sensory properties of food because of their emulsifying, thickening, and stabilizing properties, there has been little interest in their use as functional dietary fibers [36].

According to the findings of this review, *H. fusiformis* contains high amounts of ash (14–40% DW). Previous studies have reported that brown seaweed contains a higher ash content (19.60–45.48%) DW than red and green seaweed and most terrestrial plants [37,38]. A high ash content may indicate the presence of significant amounts of various mineral components [39]. Furthermore, the ash content of seaweeds is dependent on geographical, environmental, and physiological factors [40].

The moisture content of *H. fusiformis* reported in the reviewed studies exhibited a narrow range of variation, from 4.0–9.5% DW. Moisture content is an important parameter in evaluating the quality and shelf life of processed seaweed because high moisture can accelerate microbial activity and spoilage [41]. The amount of water in food or animal feed affects its usability and quality, such as texture, taste, appearance, and stability. Therefore, moisture content is critical in the utilization of seaweeds in several industries, including the food, chemical, and pharmaceutical industries [42].

#### 3.1.2. Major Minerals and Trace Elements

Seaweeds contain higher amounts of major minerals (macrominerals) and trace elements (microminerals) than terrestrial edible plants, making them a sustainable mineral source in human nutrition [37]. Edible seaweeds provide all of the essential nutrients, including calcium (Ca), magnesium (Mg), potassium (K), sodium (Na), phosphate (P), iron (Fe), copper (Cu), manganese (Mn), and zinc (Zn). Major minerals are constituents of vital cellular components, such as proteins and nucleic acids. Trace elements are defined as chemical elements contained in natural materials that are needed by humans in small amounts (<100 μg/g). Both major minerals and trace elements play important roles in biochemical reactions in living organisms in minute quantities [43]. The mineral profile of *H. fusiformis* is shown in Table 2.

As shown in Table 2, Ca was the most abundant major mineral (0.87–1.17% DW) in *H. fusiformis*, followed by Mg (0.01–0.63% DW), K (0.32–1.14% DW), Na (0.16–0.83% DW), and P (0.01% DW) [21,22]. Overall, the values reported here are in accordance with previous findings for brown seaweed [27,44]. The calcium content of *H. fusiformis* was higher than that of terrestrial foods, such as whole milk (115 mg/100 g), brown rice (110 mg/100 g), peanuts (60.0 mg/100 g), and bananas (6 mg/100 g) [45]. Among the trace elements examined in this review, Fe (14.3–47.6% DW) was the most abundant microelement, followed by Cu (0.7% DW), Mn (1.7% DW), and Zn (1.5–1.6% DW) [21,22]. The values reported here were consistent with those of previous studies on brown seaweed mineral content [27].

#### 3.1.3. Polysaccharide

As previously mentioned, brown seaweeds contain high levels of polysaccharides, such as fucoidan, alginate, and laminaran. Polysaccharides extracted from *H. fusiformis* were characterized based on their molecular weight, sulfate content, uronic acid, total carbohydrates, and neutral sugar components (Table 3).

Polysaccharides are polymers of simple sugars (monosaccharides) composed of repeating units linked together by glycosidic bonds [56]. Polysaccharides account for 40–50% of the dry matter of seaweed cell walls [57]. However, it should be noted that the biosynthesis of polysaccharides in seaweeds is influenced by both environmental and ecological conditions. Different possibilities exist for monosaccharides linked by glycosidic bonds (α or β, 1 → 3, 1 → 4, 1 →) [27]. The monosaccharide sequences present in *H. fusiformis* were neutral (Fuc, Rha, Glc, Man, Ara, Xyl, Gal, Fru) and acidic (Fuc, Rha, Glc, Man, Ara, Xyl, Gal, Fru) (GlcA, ManA, GalA, GulA), which was consistent with previous reports that monosaccharide sequences can be neutral, acidic, or hexosamines [58,59]. Additionally, it has been reported that these polymers can be both linear (alginate, and cellulose) and branched (fucoidans, and sulfated galactans) [60]. 

**Figure 4:**
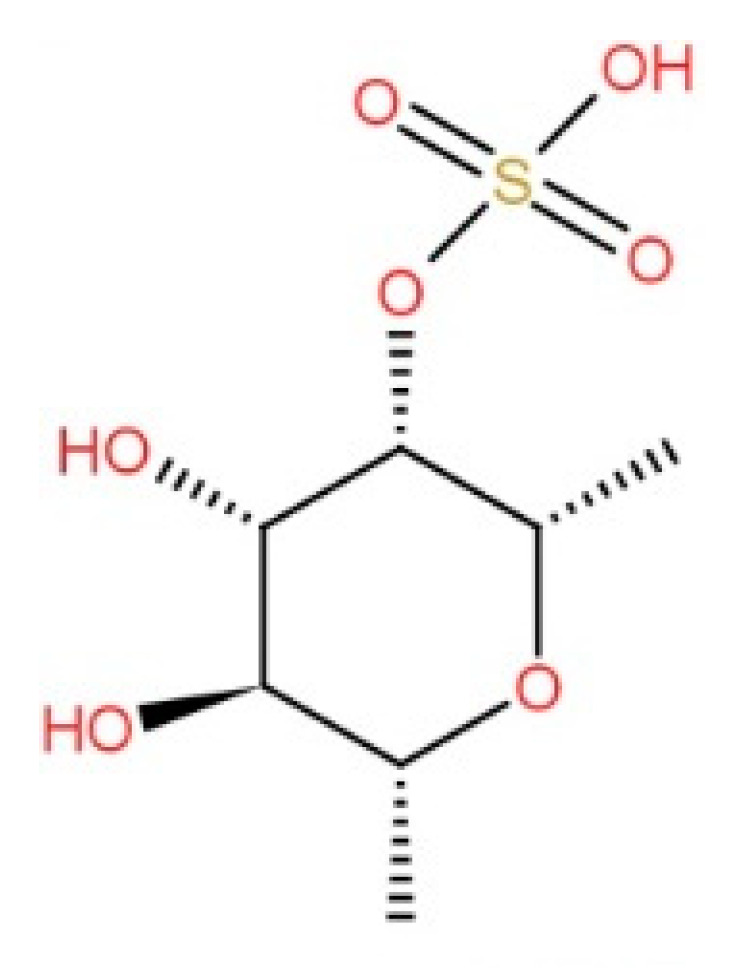
Basic chemical structure of fucoidan.

Among polysaccharides, fucoidans have been extensively studied because of their biological activities, including antioxidant, anticoagulant, antithrombotic, antiproliferative, antitumor, anticancer, immunomodulatory, anti-inflammatory, antibacterial, and antidiabetic activities [61,62]. Fucoidans are a type of sulfated polysaccharide with extremely variable molecular weights, and are commonly found in brown seaweed extracts [63]. According to previous studies, the molecular weight of fucoidan in *H. fusiformis* is between 24 and 299 kDa [9,49,51,52]. The basic chemical structure of fucoidan is shown in Figure 4.

Fucoidans are typically divided into two types: the first (I) is made up of repeating (13)-L-fucopyranose units, whereas the second (II) alternates repeating (13) and (14)-L-fucopyranose units, which in either case may be substituted with sulfate or acetate and/or have side branches containing fucopyranoses or other glycosyl units, such as glucuronic acid [64]. Fucose is the most abundant monomer of *H. fusiformis* fucoidan, but it also contains galactose, mannose, xylose, and glucuronic acid residues [46,47,53]. These components could be contaminated by other polysaccharides or genuine substitutions on fucoidan molecular entities [64]. Nonetheless, seaweed contains a number of polysaccharides that have been studied in numerous scientific articles but are yet to be commercialized.

#### 3.1.4. Bioactive Compounds

The recent surge of interest in seaweed has been fueled by its bioactive compounds, which have potential applications in nutraceuticals and pharmaceuticals. Candidate bioactive compounds in *H. fusiformis* that can be developed for industrial applications include polysaccharides (e.g., alginate and fucoidan), polyphenols (e.g., phlorotannins), glycyrrhizin, arsenic, sterol (fucosterol, saringosterol), pigments (e.g., carotenoid fucoxanthin), and fatty acids (e.g., tetradecanoic acid, 9-hexadecenoic acid, palmitic acid, and arachidonic acid) (Table 4).

**Figure 5:**
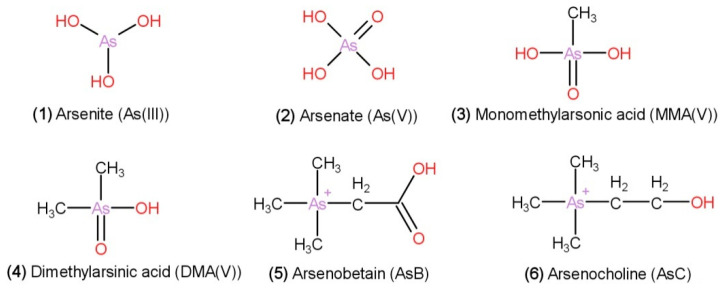
Chemical structures of arsenic compounds found in *H. fusiformis*. (**1**) Arsenite (As(III)); (**2**) arsenate (As(V)); (**3**) monomethylarsonic acid (MMA(V)); (**4**) dimethylarsinic acid (DMA(V)); (**5**) arsenobetaine (AsB); (**6**) arsenocholine (AsC).

Marine plants can accumulate arsenic from water; hence, seaweeds have higher arsenic contents than terrestrial plants. Zhao et al., (2014) investigated the total arsenic, dimethylarsinate (DMA), arsenite (As (III)), and arsenate (As (V)) contents of seaweeds and found that the total arsenic content of *H. fusiformis* was significantly higher than that of *L. japonica*, *P. yezoensis*, *U. pinnatifida*, and *E. prolifera* [68]. According to Park et al. [67], *H. fusiformis* contains inorganic arsenic: arsenite (As (III)) and arsenate (As (V)) and organic arsenic: dimethylarsinic acid (DMA), monomethylarsonic acid (MMA), arsenobetaine (AsB), and arsenocholine (AsC). The chemical structures of the arsenic compounds in *H. fusiformis* are shown in Figure 5. Generally, inorganic arsenic is more toxic than organic arsenic, and arsenite (As(III)) toxicity is 60 times higher than that of arsenate (As(V)) [73]. The LD_50_ values for arsenite, arsenate, MMA, DMA, AsC, and AsB, according to the United States Environmental Protection Agency (EPA), are 15–42, 20–200, 700–1800, 1200–2600, 6500, and 10,000 mg/kg, respectively [74]. LD_50_ is a statistically derived concentration that is expected to cause death in 50% of animals within a particular period of time [75]. Boiling *H. fusiformis* at 90 °C and soaking in 2% NaCl solution reduced the inorganic arsenic intake by consumers [67]. Furthermore, temperature was found to be a significant factor in the removal of inorganic arsenic from *H. fusiformis* using an aqueous extraction method. The optimal removal conditions for inorganic arsenic were a pH of 4, a temperature of 50 °C, a removal time of ≥8 h, a solid (dry)/liquid ratio of 1:40 (*m*/*v*), and twice extraction [22]. 

**Figure 6:**
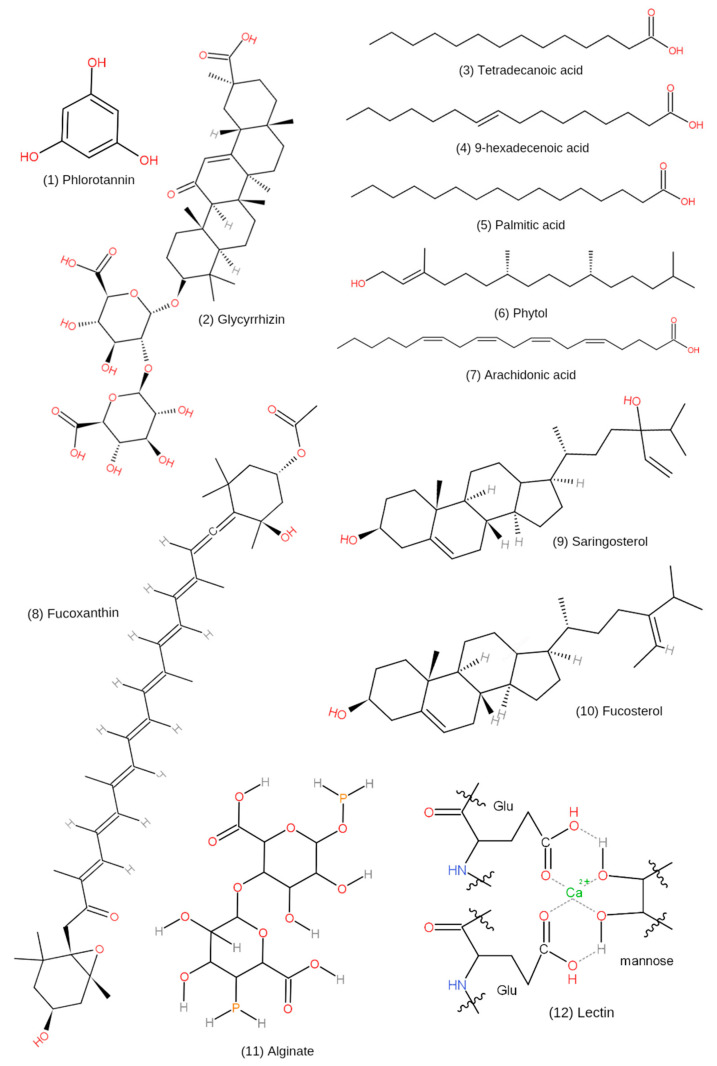
The chemical structures of bioactive compounds in *H. fusiformis*. (**1**) phlorotannins; (**2**) glycyrrhizin; (**3**) tetradecanoic acid; (**4**) palmitic acid; (**5**) 9-hexadecenoic acid; (**6**) phytol; (**7**) arachidonic acid; (**8**) fucoxanthin; (**9**) saringosterol; (**10**) fucosterol; (**11**) alginate; (**12**) lectin.

Yang et al. [69] discovered that the main components of the lipid-soluble subfraction of the *H. fusiformis* functional oil (HFFO) were tetradecanoic acid (11%), palmitic acid (37.80%), 9-hexadecenoic acid (2.67%), phytol (33.21%), and arachidonic acid (15.32%) (Figure 6). These components have anti-neuroinflammatory properties that can help prevent Alzheimer’s disease [69]. Generally, oil extracts from terrestrial plants have been shown to possess several biological properties, including anti-inflammatory, neuroprotective, and antioxidant properties, as well as the ability to increase the bioavailability of other drugs [76]. However, studies on the essential and functional oils in macroalgae are limited and may represent a source of pharmacologically active compounds.

Wagle et al. [65] investigated the activities of glycyrrhizin isolated from *H. fusiformis*, including its metabolites, 18α- and 18β-glycyrrhetinic acid, in Alzheimer’s disease prevention. Glycyrrhizin, commonly known as glycyrrhizic acid (GLR) or licorice, is a saponin molecule made up of glycyrrhetic acid, a triterpenoid aglycone, and glucuronic acid disaccharide. GLR is frequently extracted using an ethanol solution at high temperatures [77]. GLR has been permitted for use as a food additive in the United States since 1985 and is generally recognized as safe (GRAS). GLR and GLR-containing extracts from three plants, *Glycyrrhiza glabra*, *Glycyrrhiza uralensis Fisch.*, and *Glycyrrhiza inflata* Bat., have previously been studied for their characteristics and applications. Furthermore, GLR has been extensively explored in biology and medicine because of its wide range of pharmacological properties, including anti-inflammatory, antioxidant, anti-allergenic, antibacterial, antiviral, antiparasitic, and anticancer activities [78,79,80]. Based on these results, there is a need for studies to examine the pharmacological properties of GLR present in seaweeds.

Phlorotannins are polyphenols found in brown seaweeds, formed from the oligomerization and decoupling of phloroglucinol (1,3,5-trihydroxybenzene) units, and biosynthesized via the acetate–malonate pathway, commonly known as the polyketide process. Their molecular weight is between 126 kDa and 650 kDa, and their content in dried brown seaweeds ranges from 0.5–2.5% [16,81,82]. According to Li et al. [78] and Liu et al. [4], *H. fusiformis* contains 88.48 ± 0.30 mg phloroglucinol equivalents (PGE)/100 mg of the phlorotannin extract. A total of 42 chemicals with different molecular weights were discovered and tentatively characterized in *H. fusiformis*, among which, fuhalol-type phlorotannins were the most abundant [83]. The relative abundance of phlorotannins in *H. fusiformis* and its biological activity has stimulated considerable research into their potential use in several therapeutics.

Additionally, *H. fusiformis* contains fucosterol, a phytosterol with a non-polar component isolated from methanol extracts [71]. The total fucosterol concentrations in normal *H. fusiformis* extract (NH) and modified *H. fusiformis* extract (EH) were 0.249 and 1.067 mg/g, respectively [70]. Furthermore, fucosterol is an additional liver X receptor (LXR) agonist that may also play a role in the gene expression profile resulting from *H. fusiforme* supplementation [84]. Liver X receptors (LXRs), LXRα (NR1H3), and LXRβ (NR1H2) are nuclear receptors that control the metabolism of a variety of essential lipids, such as cholesterol and bile acids [85]. Therefore, further research on the biological activity of *H. fusiformis* is necessary.

Saringosterol is a non-polar sterol found in algae and is derived from methanol [6,71]. Studies have examined the chemical structures of *H. fusiformis* sterols, 24S-saringosterol and 24R-saringosterol [6,86]. 24S-saringosterol was more effective than 24R-saringosterol in LXR-mediated transactivation. 24S-saringosterol is a naturally occurring cholesterol-lowering substance that acts as a selective LXR agonist [9,86].

Fucoxanthin (Fx) is an allenic carotenoid extracted from edible brown seaweeds [87]. Carotenoid in terrestrial plants is predominantly found as β-carotene and lycopene [88]. Unlike carotenoid in terrestrial plants, Fx is a xanthophyll with a unique structure that includes an uncommon allenic link and a 5,6-monoepoxide in its molecule [89]. The Fx found in *H. fusiformis* has previously been categorized as a non-polar component isolated from methanol extracts [71]. Moreover, Dai et al. [90] reported that extracts of the fucoxanthin-rich fraction (FxRF) from *H. fusiformis* consisted of five pigments: Fx, chlorophyll-a, β-carotene, cis-fucoxanthin, and pheophytin-a. These pigments have also been found in other brown seaweeds. Since seaweeds have low Fx contents (0.02–0.58% fresh weight), obtaining sufficient quantities of Fx for commercial applications is a significant challenge [91]. However, FxRF extracts, which contain Fx and other similar bioactive pigments, are easily obtained [8]. 

Another group of bioactive compounds present in *H. fusiformis* are alginates. Alginates are linear block co-polymers composed of a 1,4-linked β-D-mannuronic acid (M) with a 4C1 ring conformation and an α-L-guluronic acid (G) with a 4C1 conformation, both in the pyranose conformation and present in varying amounts in the polymer structure [92]. Natural alginates have no regular repeating sequences, and the monomers can be arranged in homogeneous blocks of varying lengths (G-, M-, and MG-blocks) or in random patterns [93]. Cong et al. [93] demonstrated that *H. fusiformis* alginates were rich in M blocks and the average molecular weights of 04S2P-S and commercial alginate (Alg-S) were 55.5 kDa and 557 kDa, respectively. Sulfation modification of Alg-S resulted in higher molecular weights. Moreover, the polymer composition, as measured by the M/G ratio, particularly the length of the G-block, is critical in determining the physicochemical properties of alginate [94]. In the presence of divalent cations such as calcium, barium, and strontium, G-blocks can create strong hydrogels via coordination of the divalent cations in cavities formed by two contiguous G-blocks [93].

According to the literature, *H. fusiformis* contains lectins (HFL; molecular weight, 16.1 kDa), which are made up of monosaccharide units such as glucose, galactose, and fucose linked by N-glucosidic bonds [72]. Lectins are glycoproteins that are known for their aggregation and high specificity in binding to carbohydrates without initiating modifications via associated enzymatic activation [95]. Compared to other plant-derived lectins, lectins derived from algae have not been well characterized [96]. However, algal lectins have been shown to have mitogenic, cytotoxic, antibacterial, antinociceptive, anti-inflammatory, antiviral (HIV-1), platelet aggregation, and anti-adhesion properties [97]. The chemical structures of bioactive compounds in *H. fusiformis* are shown in Figure 6.

### 3.2. Pharmacological Properties

*H. fusiformis* extract has been studied by modern researchers because of its long history of use and bioactive compounds, making it a promising species for pharmacological applications. Evidence of the pharmacological properties of seaweed is described in this article.

#### 3.2.1. Antibacterial Activity

Studies on the antibacterial activity of *H. fusiformis* against selected human pathogens such as *Escherichia coli*, *Staphylococcus aureus*, *Bacillus subtilis*, *Enterobacter aerogenes*, *Shewanella* sp., *C. vilaceum*, *A. hydrophilia*, *V. parahaemolyticus*, and *P. aeruginosa* have been reported (Table 5).

Wu et al. [98] investigated the antibacterial activity of *H. fusiformis* extract. In vitro studies of seaweed phenols showed that the methanolic extract of *H. fusiformis* from Zhoushan and Mazu, exhibited high antibacterial activity against *E. coli*. Furthermore, the ethanolic extract of *H. fusiformis* from Naozhou showed high antibacterial activity against *B. subtilis* [98]. However, it should be noted that the phenolic compositions of seaweeds is influenced by several factors, including genetic and environmental factors [100].

Furthermore, in vitro antimicrobial studies showed that phlorotannin extracted from *H. fusiformis* exhibited anti-quorum sensing (QS) activity against *Chromobacterium violaceum* by reducing the production of the purple pigment. Additionally, phlorotannin reduces virulence factor production and biofilm formation. Moreover, phlorotannin can reduce mortality caused by *Pseudomonas aeruginosa* infection in *Caenorhabditis elegans* in vitro [99]. However, clinical studies on the antibacterial activity of *H. fusiformis* have not been conducted.

#### 3.2.2. Antioxidant Activity

The defense mechanism of organisms against free radical attack is mediated by antioxidants [101]. Antioxidants possess free radical scavenging properties, which can delay and ameliorate cell damage. There have been several studies on the antioxidant activity of *H. fusiformis* in different in vitro models, such as sheep erythrocytes [72], Vero cells [46,102], liver tissue [9,103], and RAW 264.7 macrophages [4], and in in vivo models, such as zebrafish embryos and mice [46,102,103,104] (Table 6). 

Wu et al. [72] confirmed the presence of a thyroglobulin-binding lectin in *H. fusiformis* using the hemagglutination inhibition test. HFL showed free radical scavenging activity against hydroxyl, DPPH, and ABTS+ radicals in sheep erythrocytes. Based on these indicators, it was concluded that *H. fusiformis* has antioxidant properties. These findings are new because algal lectins have really been reported to possess antioxidant activity, especially brown seaweed [72]. 

Fucoidan is a sulfate-rich polysaccharide complex found in seaweeds and has antioxidant properties. Fucoidan induces apoptosis via a mitochondria-mediated pathway [46,102]. This evidence is reinforced by a decrease in the levels of ROS and cleaved caspase-3 (cell death regulator) and an increase in the cell viability against azobis (2-amidinopropane) dihydrochloride (AAPH)-induced Vero cells at all concentrations (18.75, 37.5, 75, and 150 μg/mL) in vitro. Fucoidan also reduced ROS generation and lipid peroxidation in zebrafish embryos in vivo [102]. Furthermore, fucoidan can increase the survival rate and decrease the heart rate of zebrafish, indicating a protective effect against H_2_O_2_-induced damage and heart-beating disorder. [46].

The polysaccharide content in *H. fusiformis* showed protective effects against free radicals via upregulation of the Nrf2 signaling pathway. Moreover, they can significantly reduce the malondialdehyde (MDA) level and elevation of hepatic superoxide dismutase (SOD) activity in vivo [9,103,104]. Additionally, phenolic compounds and fucosterol stimulate antioxidant enzymes against free radicals, thus prolonging the lifespan of *C. elegans* [4,105]. These findings may be beneficial in the development of pharmaceutical drugs.

#### 3.2.3. Anticancer and Antitumor Activity

Several studies have reported that ethanol extracts of *H. fusiformis*, fucoidan, and alginate isolated from *H. fusiformis* possess anti-apoptotic effects [7,10,106] (Table 7).

Apoptosis, or programmed cell death, is tightly controlled at the gene level, resulting in the orderly and efficient removal of damaged cells, such as those that occur after DNA damage or during development [110]. Apoptosis may be caused by both internal and external signals, such as genotoxic stress or the binding of ligands to cell surface death receptors [111]. Cancer and tumors are characterized by the downregulation of the apoptotic cell death machinery [112].

Recent studies have shown that the ethanol extract of *H. fusiformis* has potential anticancer activity. The mechanism is mediated by activation of the intrinsic and extrinsic apoptotic pathways; thus, ROS-dependent inactivation of PI3K/Akt signaling through apoptosis was induced in B16F10 mouse melanoma cells [106]. Similar results showed that the ethanol extract of *H. fusiformis* markedly suppressed the growth of PC3 cells by regulating the ROS-dependent pathway [107]. Moreover, fucoidan potentially exhibits protective activity against cell-induced apoptosis in Hep3B cells and Chang liver cells in vitro, and suppressed cell death and ROS production in zebrafish embryos in vivo [7,102,108]. Further studies indicated that fucoidan may also inhibit lung cancer cells both in vitro and in vivo by interfering with VEGF-induced angiogenesis. Fucoidan was able to block the VEGFR2/Erk/VEGF signaling pathway in HMEC-1 and inhibit cancer cell growth in mice, while demonstrating its anti-angiogenic activity [53]. Additionally, Chong et al. [10] compared alginate (Alg-S) and alginates from other brown algae (04S2P-S) and found that Alg-S had a high anti-angiogenic effect on HMEC-1 cells. Among the five different tumor cells, 04S2P-S exhibited strong antitumor activity against Bel7402 only, whereas Alg-S possessed antitumor activity against three tumor cell lines, including Bel7402, SMMC7721, and HT-29 cell lines [10]. Furthermore, Lee et al. [109] demonstrated that the *H. fusiformis* solvent-partitioned fractions inhibits MMP activity and intracellular MMP pathways by regulating TIMP expression in HT1080 human fibrosarcoma cells. The antitumor effect of *H. fusiformis* is strengthened because it has a high level of cytotoxicity against HepG2 cells. *H. fusiformis* significantly inhibited tumor growth in nude mice in vivo [52]. These findings indicated that *H. fusiformis* has the potential to be used as a chemopreventive and/or adjuvant chemotherapeutic drug for the treatment of cancer and tumors.

#### 3.2.4. Anti-Inflammatory Activity

Inflammation is a complex defense mechanism that seeks to restore normal cell structure and function in response to microbial and endotoxin infections, wounds, and irritants [113]. Various studies have been published on the anti-inflammatory potency of *H. fusiformis* (Table 8).

Fucoxanthin, a bioactive compound in seaweed, decreased cell viability by 60% and cytokine levels in macrophages and keratinocytes by inhibiting the MAPK pathway, indicating its therapeutic effect during inflammation. Moreover, in particulate matter (PM)-exposed zebrafish embryos, fucoxanthin significantly reduced the expression levels of factors involved in inflammatory responses and cell death, including NO and reactive oxygen species [8]. Previous reports have shown that *H. fusiformis* extract decreases iNOS expression and NF-κB translocation, and increases the activation of MAPKs and STAT1 phosphorylation [114].

The release of various inflammatory mediators has been linked to the progression of several inflammatory diseases, including atopic dermatitis and allergic rhinitis. Atopic dermatitis is a chronic inflammatory skin disease characterized by immunoglobulin E (IgE) antibodies and helper T cells that contain type 2 (Th2) cytokines associated with cutaneous hyper-reactivity to environmental stimuli, triggering inflammation [118]. Furthermore, *H. fusiformis* phlorotannin remarkably inhibited the production of pro-inflammatory mediators, including nitric oxide (NO), interleukin-6 (IL-6), prostaglandin E2 (PGE2), and tumor necrosis factor-α (TNF-α) [66]. Oral administration of fucosterol (200 mg/kg weight/day) extracted from *H. fusiformis* was reported to reduce systemic inflammation effects in 2,4-dinitrochlorobenzene (DNCB)-induced AD-like lesions in NC/Nga mice by regulating the Th1/Th2 immune balance [115]. Ho et al., demonstrated that the final fraction (F2′) from *H. fusiformis* contained a higher proportion of butanoic acid, which could be a strong candidate for anti-atopic dermatitis. They evaluated induced AD damage in male BALB/c mice and found that the dephosphorylation of nuclear factor of activated T cells (NFAT) was inhibited in an electrophoretic mobility shift assay. As a result, cytokines produced by helper T cells, such as interleukin-2,-4, and interferon-γ, were significantly reduced while the cells were activated [116]. Additionally, *H. fusiformis* treatment in mouse models challenged with allergic rhinitis inflammation showed anti-inflammatory and anti-allergic effects by suppressing T-helper type 2 cytokine production (IL-13) both locally and systemically; goblet cell hyperplasia OVA-specific IgE formation, and eosinophilic infiltration were all reduced [117]. These properties of *H. fusiformis* may be beneficial for the treatment of atopic and allergic diseases.

#### 3.2.5. Photoprotective Activity

Plants and other autotrophic organisms are known to have photoprotective mechanisms, which are biochemical processes that protect against sun radiation by preventing the skin from oxidative stress. Sun radiation contains a variety of electromagnetic spectra, including UV rays, which can cause skin cancer from excessive exposure [119]. Numerous studies on the photoprotective potential of *H. fusiformis*-derived compounds against ultraviolet irradiation have been published [23,120,121,122] (Table 9).

Wang et al. [120] demonstrated that fucoidan treatment in HDF cells exposed to UVB (50 mJ/cm^2^) exhibited protective effects in an in vitro experiment. Its effects included cell death reduction due to the scavenging of intracellular ROS, collagen synthesis, inhibition of intracellular collagenase, suppression of MMP, PGE2, and pro-inflammatory cytokine expression via the NF-B, AP-1, and MAPK pathways. Furthermore, in vivo experiments have shown that seaweed extracts can reduce ROS scavenging and decrease cell death in zebrafish larvae induced with UVB photodamage by reducing lipid peroxidation and inflammatory responses [50,120]. Additionally, fucosterol treatment significantly reduced UVB-induced expression of MMP-1, IL-6, p-c-Jun, and p-c-Fos and increased type I procollagen expression in NHDF cells [23]. In a previous study, fucoidan significantly reduced ROS levels, enhanced cell viability, and suppressed UVB-induced apoptosis in ultraviolet (UV) B-irradiated human keratinocytes (HaCaT cells [122]. Additionally, *H. fusiformis* extracts exhibited anti-melanogenesis effects in RAW 264.7 and B16F10 cell lines induced by ultraviolet irradiation, and decreased melanin biosynthesis by inhibiting α-MSH-stimulated melanogenesis [121,122]. Furthermore, seaweed extract reduced oxidative stress in UVB-exposed hairless Kun Ming mice by increasing superoxide dismutase (SOD) and catalase (CAT) activities, and decreasing ROS, malondialdehyde (MDA) equivalents, and matrix metalloproteinase (MMP)-1 and 9 levels [54]. These data show that *H. fusiformis* can be used as a skin-protective agent.

#### 3.2.6. Neuroprotective Activity

The term “neuroinflammation” refers to an inflammatory response that is centralized within the brain or spinal cord. Some studies have examined the effect of *H. fusiformis* extract on neuroinflammation (Table 10).

The production of cytokines, reactive oxygen species (ROS), chemokines, and secondary messengers mediate this inflammation [126]. However, uncontrolled neuroinflammatory responses cause neuronal damage including Huntington’s, Alzheimer’s, and Parkinson’s disease. *H. fusiforme* was reported to contain a neuroprotective compound: 5-hydroxy-3,6,7,8,3′4′-hexamethoxyflavone (5HHMF). 5HHMF significantly inhibited lipopolysaccharide (LPS)-stimulated NO production by suppressing the expression of inducible NO synthase (iNOS) in BV2 microglia [124] and inhibiting pro-inflammatory cytokines, as well as the expression of NF-κB activation [123].

Several studies on the effects of *H. fusiformis* extract against Alzheimer’s disease have reported that saponin and glycyrrhizin and its metabolites (18β-glycyrrhetinic acid) inhibited β-site amyloid precursor protein cleaving enzyme 1 (BACE1). Overall, glycyrrhizin was 2- and 11-fold more effective than GLR and 18a-glycyrrhetinic acid, respectively [65]. The polysaccharide, SFPS65A, extracted from *H. fusiformis*, was reported to enhance the cognitive abilities of drug-treated mice in memory loss models [47]. Furthermore, a sterol (24-(S)-saringosterol) extracted from *H. fusiformis* enhanced cognition and alleviated disease by selectively activating liver X receptor β when Alzheimer’s disease mice were fed with *H. fusiformis* or its extract [125]. Furthermore, the *H. fusiformis* functional oil (HFFO) inhibited acetylcholinesterase (AChE), NO production, and reduced the ROS levels in BV-2 cells (mouse microglia) [69].

#### 3.2.7. Antidiabetic Activity

An antidiabetic substance is defined as any substance that can help patients with diabetes by controlling blood sugar levels in the body. A number of studies regarding the antidiabetic effects of *H. fusiformis* have been conducted in vitro and in vivo [49,71,127,128] (Table 11).

Polyphenol suppressed α-glucosidase activity in C57BL/6N muscle tissue (mice) in an in vitro experiment [127]. The result of an in vivo assay showed that there was an increase in muscle glucose uptake and insulin signaling-related proteins in C2C12 myotube cells (mice) fed a high-fat diet supplemented with 5% *H. fusiformis* for 16 weeks [127]. Polysaccharides of *H. fusiformis* possess significant hypoglycemic and hypolipidemic activities [49] and enhanced glycogen storage in the liver and skeletal muscle [128] in type 2 diabetic rats. Moreover, Seong et al., investigated the non-polar constituents of *H. fusiformis* that could potentially suppress glucose absorption via inhibition of the α-glucosidase enzyme in the digestive organs and can also stimulate the insulin signaling pathway in HepG2 cells via inhibition of the PTP1B enzyme in insulin-sensitive organs [71]. Cheng et al. [55] reported that treatment with fucoidan extracts of *H. fusiformis* reduced fasting blood glucose (FBG), food and water intake and normalized the histopathological parameters of heart and liver functions in steptozotocin (STZ)-induced mice. These findings indicate that seaweed can serve as an alternative functional food that can complement the management of diabetes in the future.

#### 3.2.8. Immunomodulatory Effects

Immunoprecipitation refers to interventions that cause specific changes to the immune system by increasing (immunostimulatory) or decreasing (immunosuppressive) antibody synthesis, regardless of the body’s health or nutritional status. Vitamin A, C, D3, β-carotene, and other minerals found in plants may act as immunomodulators [129,130]. Several investigations have reported that *H. fusiformis* extracts, including polysaccharides, fucoidan, and fucosterol, may potentially regulate the immune system [11,51,70] (Table 12).

An in vitro study on bone marrow-derived dendritic cells (DCs) demonstrated that *H. fusiformis* extract can regulate the activation and maturation of DCs. Furthermore, there was an increase in splenic DC maturation and CD8+ T cell activation in mice treated with *H. fusiformis* for 3 days [131]. Additionally, the in vitro immunomodulatory activities of *H. fusiformis* polysaccharides on murine macrophages and splenocytes were investigated. In RAW 264.7 cells and splenocytes, polysaccharides exhibited potential macrophage-stimulating effects, such as NO production and increased pro-inflammatory cytokines [11].

Park et al. [70] investigated differences in the immunostimulatory activities of enzyme-modified *H. fusiforme* extracts (EH) and normal *H. fusiforme* extracts (NH) and found that EH increased TNF-secretion, NO production, and phagocytotic activity. Additionally, *H. fusiforme* extracts have been shown to increase splenocyte proliferation and restore cytokine levels in vivo.

The immunostimulatory activities of the *H. fusiformis* aqueous extract (HFAE) showed a dose-dependent manner; HFAE stimulated RAW 264.7 macrophages to produce cytokines such as NO, TNF-α, IL-1β, and IL-6, and increased their mRNA expression. Moreover, stimulated RAW 264.7 macrophages secreted NO by inducing iNOS protein expression. Furthermore, HFAE promoted the proliferation and induction of IL-2 and TNF-α protein expression in spleen cells [132]. A similar study by Chen et al., showed that polysaccharides can upregulate cytokine production and activate the NF-κB signaling pathway via CD14/IKK and P38 Axes in RAW264.7 cells [51].

#### 3.2.9. Osteoprotective Activity

Osteoarthritis (OA), a widely known type of arthritis, is a degenerative joint disease characterized by joint pain and swelling caused by the gradual loss of articular cartilage. Age, sex, family history, joint stress, and obesity are major risk factors for OA. It can reduce the quality of life by interfering with daily activities and causes movement disorders [133]. Bioactive compounds contained in plants and seaweed are thought to be able to prevent osteo-disease and act as osteoprotective agents. Several studies have demonstrated the osteoprotective activity of *H. fusiformis* both in vivo and in vitro, in mice (Table 13).

Kwon et al. [12] reported that a 20% ethanol extract of *H. fusiformis* inhibited anabolic, catabolic, and genetic factors of osteoarthritis in cartilage cells and osteoarthritis-induced SD rats both in vitro and in vivo. Additionally, Lee et al. [13] reported decreased bone loss, reduced articular cartilage inflammation, and increased cytokine levels in fucoidan-treated rats previously injected with monosodium iodoacetate (MIA).

*H. fusiformis* showed beneficial osteoprotective effects in vitro by enhancing osteoblast differentiation via ALP and BMP-2 stimulation, and inhibiting osteoclast differentiation to prevent osteo-disease. Furthermore, there was an increase in BMP2a and 2b levels and bone regeneration in vivo in mouse C2C12 cells, bone marrow cells (male ICR mice), zebrafish embryos, and ovariectomized (OVX) mice [134]. Previous studies have indicated that fucosterol isolated from *H. fusiformis* increased the proliferation of MG63 cells in the treatment of bone-resorbent metabolic bone disorders, such as osteoporosis and periodontitis [6].

#### 3.2.10. Gastroprotective Activity

Gastrointestinal disorders, including gastric mucosa, gastritis, and gastric and peptic ulcers, can be caused by organ damage caused by toxic agents. Ethanol is a common substance that can cause gastrointestinal disorders, which is commonly abused by humans, causing harm to several organs. Polysaccharides isolated from *H. fusiformis* have shown gastroprotective effects against several gastrointestinal diseases based on the existing literature (Table 14).

Several pharmaceutical products have been developed to treat gastrointestinal diseases such as ulcer hemorrhage and perforation [136], and polysaccharides extracted from *H. fusiformis* have been shown to exhibit protective effects against ethanol-induced cellular damage in vitro and in vivo. Furthermore, polysaccharides extracted from *H. fusiformis* reduced the total glutathione (GSH) levels and enhanced Jun N-terminal kinase (JNK) phosphorylation in IEC-6 cells exposed to alcohol [24,135]. Similarly, Sun et al., (2019) reported that ethanol-induced gastric ulcer was inhibited in rats treated with *H. fusiformis* polysaccharide extracts, which modified various molecules involved in the initiation of gastric ulcers [48]. These results suggest that polysaccharides extracted from *H. fusiformis* could be a potential therapeutic agent for gastrointestinal diseases.

## 4. Conclusions

Based on the findings of this review, it can be concluded that *H. fusiformis* has great potential as a source of high-value compounds because of its nutritional and pharmacological properties. Although *H. fusiformis* has been used since ancient times in traditional medicine and for food, scientific studies validating and confirming its effects are still lacking. *H. fusiformis* contains higher amounts of major minerals (macronutrients) and trace elements (micronutrients) than terrestrial edible plants. Furthermore, some bioactive compounds isolated from *H. fusiformis*, including polysaccharides (e.g., alginate and fucoidan), polyphenols (e.g., phlorotannins), glycyrrhizin, arsenic, sterol (fucosterol, and saringosterol), pigments (e.g., carotenoid fucoxanthin), and fatty acids (e.g., tetradecanoic acid, 9-hexadecenoic acid, palmitic acid, and arachidonic acid) can be further evaluated for use as nutraceuticals and pharmaceuticals.

The nutritional and pharmacological properties of *H. fusiformis* are promising for industrial applications. Some strategies could be applied for the use of *H. fusiformis* for medicinal and nutritional purposes. The development of marine resources has challenges in terms of the supply and maintenance of the quality of nutritional value and bioactive compounds. Hence, the first strategy is to increase *H. fusiformis* production through economical, efficient, and sustainable cultivation methods. Presently, South China and Korea are the largest producers of *H. fusiformis*. The second strategy should involve the standardization of nutritional components and bioactive compounds isolated from *H. fusiformis*. The third strategy is to conduct further research on bioactive and other valuable compounds present in *H. fusiformis*. Although the number of studies on bioactive compounds in *H. fusiformis* has increased, especially regarding antioxidant and anticancer bioactivity, most studies are still in the preliminary stage.

## Figures and Tables

**Table 1:** Proximate composition of *H. fusiformis*.

Primary Metabolite (% DW)						Reference
Carbohydrates	Protein	Lipid	Fiber	Ash Content	Moisture	
40.73	18.41	nd	nd	16.63	nd	[21]
nd	12.2	1.8	11.3	14	nd	[22]
61.85 ± 3.56	12.94 ± 3.61	1.76 ± 0.07	nd	19.18 ± 0.09	4.27 ± 0.12	[23,24]
nd	10.4 0 ± 0.59	1.38 ± 0.08	nd	17.89 ± 0.05	5.71 ± 0.34	[25]
nd	9.9	1.2	nd	40	9.5	[26]

% DW, g/100 g on a dry weight basis; nd, not determined.

**Table 2:** Mineral profile of *H. fusiformis*.

Metabolite Class	Dietary Content (%DW)
Major minerals	
Ca	0.87–1.17
Mg	0.01–0.63
K	0.32–1.14
Na	0.16–0.83
P	0.11
Trace elements	
Fe	14.3–47.6
Cu	0.7
Mn	1.7
Zn	1.5–1.6

% DW, g/100 g on a dry weight basis. Adapted from Choi et al., (2014) and Zheng et al., (2013) [21,22].

**Table 3:** The polysaccharide profile of *H. fusiformis*.

Type of Polysaccharides	Mw (kDa)	Chemical Composition (%DW)				Monosaccharide Composition (Weight Ratio)	Ref.
		Total Carbohydrate	Sulfate	Uronic Acid	Protein		
Fucoidan	102.67	71.79 ± 0.56	27.22 ± 0.05	nd	nd	Fuc:Rha:Glc:Man:Ara = 79.2:2.1:0.2:18.1:0.4	[46]
Crude polysaccharide	75	97.9	9.2	51.2	nd	Fuc:Rha:Glc:Man:Xyl:Gal:GluA:ManA:GulA = 28.9:5.3:1:6.1:5.2:9.1:3.8:8.8:38.9	[9]
Fucoidan	90	67.5	17.5	41.04	5.22	Fuc:Man:Xyl:Gal:Glc:GlcA = 19.2:2.6:6.6:9.6:1.0:6.5	[47]
Crude polysaccharide	229	42.69	25.69	nd	nd	Fuc = 19.5	[48]
Crude polysaccharide	58.28 and 7.46	73.86 ± 0.85	5.17 ± 0.57	32.62 ± 1.43	0.51 ± 0.08	Fuc:Rha:Glc:Man:Gal:GlcA = 43.9:2.5:6.5:16.3:18.7:12.1	[49]
Fucoidan	30–50	nd	11.60	nd	nd	Fuc:Rha:Glc:Man:Ara:Xyl:Gal = 61.5:1.2:0.7:5:0.1:7.1:24.5	[13]
Crude polysaccharide	nd	nd	63.56 ± 0.32	nd	nd	Fuc:Glc:Man:Xyl:Gal = 53.5:5.9:17.4:23.1	[50]
Crude polysaccharide	24	62.9	27.7	14.7	0.4	Fuc:Man:Xyl:Gal:GalA = 80.6:2.4:3.0:13.3:0.7	[51]
Crude polysaccharide	299	nd	10.74	6.48	nd	Fuc:Man:Xyl:Gal = 5.9:2.3:1.0:2.2	[52]
Fucoidan	47.5	16.8	20.8	34.6	Nd	Fuc:Man:Xyl:Gal:GlcA = 36.6:7.0:18.3:19.1:19.1	[53]
Crude polysaccharide	224	58.10 ± 2.12	9.85 ± 0.96	17.66 ± 0.54	1.01 ± 0.15	Fuc:Rha:Glc:Man:Xyl:Gal:Fru = 28.8:2.3:1.0:6.0:3.9:12.3:12.3	[54]
Fucoidan	205.8	68.33	14.55	nd	4.13	Fuc:Rha:Glc:Man:Xyl:Gal = 16.7:1.0:1.6:1.3:1.1:6.2	[55]

% DW, mg/100 g on a dry weight basis; nd, not determined; Mw, molecular weight.

**Table 4:** Bioactive compounds in *H. fusiformis*.

Chemical	Characteristics	Reference
Glycyrrhizin	Its metabolites, 18α-glycyrrhetinic acid and 18β-glycyrrhetinic acid	[65]
Phlorotannins	Total content 88.48 ± 0.30 (mg/100 mg DW)	[4]
	Total content 43.3 µg/mL	[66]
Arsenic compounds	Inorganic arsenic: arsenite [As(III)] and arsenate [As(V)], and organic arsenic: dimethylarsinic acid (DMA), monomethylarsonic acid (MMA), arsenobetaine (AsB), and arsenocholine (AsC)	[67]
	*H. fusiformis* has the highest total As content compared with that of *L. japonica*, *P. yezoensis*, *U. pinnatifida, E. prolifera*. Contains dimethylarsinate (DMA), arsenite [As (III)], arsenate [As (V)]	[68]
*H. fusiformis* functional oil (HFFO)	Its main components are tetradecanoic acid (11%), 9-hexadecenoic acid (2.67%), palmitic acid (37.80%), phytol (33.21%), and arachidonic acid (15.32%)	[69]
Fucosterol	The total concentration in normal HF extract (NH) and modified HF extract (EH) are 0.249 mg/g and 1.067 mg/g, respectively	[70]
	Non-polar components which are extracted from methanol	[71]
Saringosterol	24R-saringosterol is an uncommon sterol in algae, with potential role in the inhibition of MG63 cell proliferation	[6]
	Non-polar components which are extracted from methanol	[71]
Fucoxanthin	Comprised of five pigments: Fx, chlorophyll-a, β-carotene, cis-fucoxanthin, and pheophytin-a.	[8]
	Non-polar components which are extracted from methanol	[71]
Alginate	Rich in M blocks and the average molecular weights of 04S2P-S and commercial alginate (Alg-S) were 55.5 kDa and 557 kDa, respectively. Sulfation modification in Alg-S produced higher molecular weights	[10]
Lectin (HFL)	Molecular weights (16.1 kDa) and the monosaccharide units of HFL are glucose, galactose and fucose. HFL may be linked by N-glucosidic bonds	[72]

**Table 5:** Summary of the antibacterial activity of *H. fusiformis*.

Experimental Models	Extract or Constituent	Study Type	Microorganism	Effects	Ref.
nd	Phenolic Content	In vitro	*Escherichia coli* *Staphylococcus aureus* *Bacillus subtilis* *Enterobacter aerogenes* *Shewanella* sp.	Methanol extracts of *H. fusiformis* from Zhoushan and Mazu against *E. coli* showed moderate inhibitory activity (11–16 mm) Ethanol extract of *H. fusiformis* from Naozhou showed moderate inhibitory activity (11–16 mm) against *B. subtilis*	[98]
*Caenorhabditis elegans*	Phlorotannins	In vitro and in vivo	*Chromobacterium violaceum* *E. coli* *S. aureus* *Aeromonas hydrophilia* *Vibrio parahaemolyticus* *Pseudomonas aeruginosa*	In vitro: inhibited the anti-quorum sensing (QS) activities at 0.04858 g/mL, reduced virulence factor production and biofilm formation. In vivo: increased survival rate of *P. aeruginosa*-infected *C. elegans* to >80% during the first 4 days of treatment	[99]

nd, not determined.

**Table 6:** Summary of the antioxidant activity of *H. fusiformis*.

Experimental Models	Extract or Constituent	Antioxidant Assay	Scavenging Activity (%)	Study Type	Effects	Ref
Sheep erythrocytes	Lectin	Hemagglutination, DPPH, hydroxyl, and ABTS+	Hydroxyl: 33.65% and DPPH: 77.23%	In vitro	Showed free radical scavenging activity against hydroxyl, DPPH, and ABTS+ radicals	[72]
Vero cells and zebrafish embryos	Fucoidan	DPPH, alkyl, and hydroxyl	>80%	In vitro and in vivo	In vitro: reduced ROS level, increased cell viability, and inhibited cleavage caspase-3. In vivo: reduced ROS generation and lipid peroxidation	[102]
Vero cells and zebrafish embryos	Fucoidan	DPPH, hydroxyl, and alkyl	≤80%	In vitro and in vivo	In vitro: Reduced apoptosis. In vivo: increased the survival rate and decreased the heart rate	[46]
Liver tissues and ICR mice	Sulfated polysaccharides	DPPH and hydroxyl	100%	In vitro and in vivo	In vitro: Exhibited free radical scavenging activity and enhanced cell viability. In vivo: enhanced cytoprotective potential via upregulation of the Nrf2 signaling pathway	[9]
BALB/c mice and Liver tissues	Water-soluble polysaccharides	DPPH and hydroxyl	DPPH: >20 to ≤70% and hydroxyl: ≥20 to ≤100%	In vitro and in vivo	In vitro: showed free radical scavenging activity against hydroxyl and DPPH radicals. In vivo: reduced the MDA level and elevation of hepatic SOD activity	[103]
RAW 264.7 macrophages	Phenolic compounds	DPPH	<100%	In vitro	Protective effect against oxidant and inflammatory activity	[4]
ICR male mice	Polysaccharide	nd	nd	In vivo	Stimulated antioxidant enzymes against free radicals	[104]
*C. elegans*	Fucosterol	nd	nd	In vivo	Prolonged the lifespan of *C. elegans*	[105]

nd, not determined.

**Table 7:** Summary of the anticancer and antitumor activities of *H. fusiformis*.

Experimental Models	Extract or Constituent	Study Type	Optimum Dose	Effects	Ref.
B16F10 mouse melanoma cells	Ethanol extract	In vitro	400 µg/mL	Activated the intrinsic and extrinsic apoptotic pathways and the ROS-dependent pathway inactivated the PI3K/Akt signaling	[106]
Human prostate cancer PC3 cells	Ethanol extracts	In vitro	100 µg/mL	Suppressed PC3 cells growth and apoptosis via regulating a ROS-dependent pathway	[107]
Hep3B human liver cancer cell line	Fucoidan	In vitro	50 µg/mL	Reduced Hep3B cell growth	[7]
Bel7402, SMMC7721, Huh7, HT-29 and Caco-2 cells	Alginate	In vitro	nd	Inhibited the cell growth of Bel7402, SMMC7721, and HT-29 cell lines	[10]
Chang liver cells and zebrafish embryos	Fucoidan	In vitro and in vivo	100 µg/mL	In vitro: increased the viability of cells, decreased ROS levels, and inhibited apoptosis. In vivo: suppressed cell death and ROS production	[108]
Human microvascular endothelial cells (HMEC-1) and mice	Fucoidan	In vitro and in vivo	nd	Interfered VEGF-induced angiogenesis	[53]
HT1080 Human fibrosarcoma cells	*H. fusiformis* crude extract	In vitro	50 µg/mL	Inhibited MMP activity and intracellular MMP pathways via regulation of TIMP expression	[109]
Human hepatocellular carcinoma (HepG2) cells and mice	Polysaccharide	In vitro and in vivo	2000 µg/mL	In vitro: demonstrated a high level of cytotoxicity against HepG2 cells. In vivo: significantly decreased the tumor growth	[52]

nd, not determined.

**Table 8:** Summary of the anti-inflammatory activity of *H. fusiformis*.

Experimental Models	Extract or Constituent	Study Type	Optimum Dose	Effects	Ref.
RAW264.7 macrophages, HaCaT keratinocytes, and zebrafish embryos	Fucoxanthin	In vitro and in vivo	100 µg/mL	In vitro: decreased cell viability and cytokine levels. In vivo: decreased nitric oxide (NO), ROS, and cell death	[8]
RAW 264.7 cells	HF extract	In vitro	250 µg/mL	Inhibited iNOS expression, NF-κB translocation, activated MAPKs, and STAT1 phosphorylation	[114]
RAW 264.7 cells and NC/Nga male mice	Fucosterol	In vitro and in vivo	50 µg/mL	In vitro: reduced NO production. In vivo: regulated the Th1/Th2 immune balance and reduced systemic inflammation	[115]
Male BALB/c mice	Ethyl acetate (EA) extract	In vitro and in vivo	100 µg/mL	In vitro: inhibited activation of T cell activation by eliminating NFAT dephosphorylation. In vivo: inhibited activation of T cell activation by suppressing Th cell-dependent cytokines	[116]
BALB/c mice	HF extract	In vivo	nd	Suppressed T-helper type 2 cytokine production (IL-13)	[117]
Macrophage cell line RAW 264.7	Phlorotannin	In vitro	43.3 µg/mL	Inhibited the production of pro-inflammatory mediators	[66]

nd, not determined.

**Table 9:** Summary of photoprotective activity of *H. fusiformis*.

Experimental Models	Extract or Constituent	Study Type	Optimum Dose	Effects	Ref.
Human dermal fibroblast (HDF) cells exposed to UVB (50 mJ/cm^2^) and zebrafish larvae	Fucoidan	In vitro and in vivo	≤50 μg/mL	In vitro: suppressed cell death, MMPs, PGE2, and pro-inflammatory cytokines and elevated collagen. In vivo: reduced ROS levels and inflammatory responses	[120]
RAW 264.7 cell line and B16F10 cell line	Crude sulfated polysaccharides	In vitro	100 μg/mL	Inhibited lipopolysaccharide (LPS)-induced inflammation, and reduced α-MSH-stimulated melanogenesis.	[121]
Normal human dermal fibroblasts (NHDFs)	Fucosterol	In vitro	nd	Reduced the UVB- induced expression of MMP-1, IL-6, p-c-Jun, and p-c-Fos, and increased type I procollagen expression	[23]
Human keratinocytes (HaCaT cells) and B16F10 melanoma cells	Fucoidan	In vitro	100 μg/mL	Reduced ROS levels, enhanced cell viability, suppressed UVB-induced apoptosis in HaCaT cells and inhibited melanin biosynthesis	[122]
Human dermal fibroblasts	Sulfated polysaccharides	In vitro	≤50 μg/mL	In vitro: suppressed cell death, MMPs, PGE2, and pro-inflammatory cytokines and elevated collagen. In vivo: reduced ROS levels and inflammatory responses	[50]
Kun Ming Mice	Polysaccharide	In vivo	600 mg/kg/day	Exhibited protection against UVB due to decreased oxidative stress	[54]

nd, not determined.

**Table 10:** Summary of neuroprotective activity of *H. fusiformis*.

Experimental Models	Extract or Chemical or Constituent	Study Type	Optimum Dose	Effects	Ref.
BV-2 cells (mouse microglia)	*H. fusiformis* functional oil (HFFO)	In vitro and in silico	20 μg/mL	Inhibited acetylcholinesterase (AChE) and nitric oxide (NO) production, reduced ROS levels	[69]
Cell sample	Glycyrrhizin	In vitro and in silico	nd	Inhibited BACE1 activity	[65]
Murine BV-2 microglial cells	HF extract	In vitro	2 mg/mL	Increased the NO levels. Inhibited iNOS expression, pro-inflammatory cytokines, and expression of NF-κB activation	[123]
Murine BV2 microglia	Nd	In vitro	500 ng/mL	Suppressed LPS-induced iNOS expression	[124]
Male ICR mice	Polysaccharide	In vivo	nd	Improved cognitive abilities	[47]
Male ICR mice	*H. fusiformis* or its extract	In vivo	5 µg/mL	Enhanced cognition and alleviated disease	[125]

nd, not determined.

**Table 11:** Summary of antidiabetic activity of *H. fusiformis*.

Experimental Models	Extract or Chemical or Constituent	Study Type	Optimum Dose	Effects	Ref.
C57BL/6N muscle tissue (mice) and C2C12 myotube cells (mice)	Polyphenols	In vitro and in vivo	100 µg/mL	In vitro: reduced α-glucosidase activity. In vivo: enhanced muscle glucose uptake, activated insulin signaling-related proteins	[127]
Male SD rats	Polysaccharide Extract	In vivo	nd	Improved hypoglycemic activity via restoration of insulin resistance and mitochondrial function of skeletal muscle	[49]
Male SD rats	Polysaccharide	In vivo	nd	Enhanced storage of glycogen in liver and skeletal muscle and suppressed gluconeogenesis	[128]
Human recombinant PTP1B	Methanol extract	In vitro	nd	PTP1B and α-glucosidase inhibitors	[71]
Mice	Fucoidan	In vivo	nd	Reduced fasting blood glucose levels, diet, and water intake	[55]

nd, not determined.

**Table 12:** Summary of the immunomodulatory effects of *H. fusiformis*.

Experimental Models	Extract or Constituent	Study Type	Optimum Dose	Effects	Ref.
Dendritic cells (DCs) and C57BL/6 (8 weeks) mice	HF extract	In Vitro and in vivo	5 μg/mL	In vitro: induced functional and phenotypical maturation of DCs. In vivo: activated CD8+ T cells	[131]
Murine macrophages Raw 264.7 cells and splenocytes	Polysaccharide	In vitro	1 mg/mL	Increased NO production and pro-inflammatory cytokine levels	[11]
Murine macrophages and C57BL/6 mice	Fucoidan and fucosterol	In Vitro and in vivo	100 μg/mL	In vitro: increased production of NO, secretion of tumor necrosis factor-α (TNF-α), and phagocytosis activity. In vivo: stimulated splenocyte proliferation and restored the level of cytokines	[70]
RAW 264.7 macrophages and C57/BL6 mice	Lipopolysac- charide	In vitro and in vivo	5 μg/mL	In vitro: increased cytokine expression. In vivo: regulated the immune function	[132]
RAW264.7 cells	Polysaccharide	In vitro	nd	Upregulated cytokine production and activation of the NF-κB signaling pathway	[51]

nd, not determined.

**Table 13:** Summary of osteoprotective activity of *H. fusiformis*.

Experimental Models	Extract or Constituent	Study Type	Optimum Dose	Effects	Ref.
Cartilage cells and Male SD rats	HF extract	In vitro and in vivo	600–1000 μg/mL	In vitro: reduced pro-inflammatory cytokine responses. In vivo: reduced the articular cartilage damage and development of OA	[12]
Male SD rats	Fucoidan	In vivo	100 mg/kg	Prevented OA progression by decreasing bone loss, prevented articular cartilage inflammation, and increased cytokine levels	[13]
Mouse C2C12 cells, bone marrow cells, zebrafish embryos, ovariectomized (OVX) mice, mouse calvarial bone	Polysaccharide	In vitro and in vivo	200 μg/mL	In vitro: enhanced osteoblast differentiation via alkaline phosphatase (ALP) and bone morphogenetic protein-2 (BMP-2) stimulation, suppressed osteoclast differentiation. In vivo: increased BMP2a and 2b levels, protected bone mass loss, and increased bone regeneration	[134]
MG63 cells	Fucosterol	In vitro	nd	Protective activity through bone-resorbent metabolic bone disorders	[6]

nd, not determined; OA, osteoarthritis.

**Table 14:** Summary of the gastroprotective activity of *H. fusiformis*.

Experimental Models	Extract or Constituent	Study Type	Optimum Dose	Effects	Ref.
IEC-6 cells and SD rats	Polysaccharide Extract	In vitro and in vivo	500 μg/mL	Decreased the phosphorylation of Shc and JNK	[24]
IEC-6 cells and SD rats	Polysaccharide Extract	In vitro and in vivo	300 mg/kg	In vitro: reduced phosphorylation of Shc. In vivo: reduced total glutathione levels and enhanced JNK phosphorylation	[135]
Wistar albino adult male rats	Polysaccharide	In vivo	300 mg/kg	Suppressed oxidative stress and showed anti-inflammatory and antioxidant activity	[48]

JNK, jun N-terminal kinase.

## Data Availability

Data supporting reported results are available upon request.
